# Atypical monocyte dynamics in healthy humans in response to fasting and refeeding are distinguished by fasting HDL and postprandial cortisol

**DOI:** 10.1152/ajpendo.00158.2024

**Published:** 2024-07-03

**Authors:** Ryan G. Snodgrass, Charles B. Stephensen, Kevin D. Laugero

**Affiliations:** ^1^Immunity and Disease Prevention Research Unit, Western Human Nutrition Research Center, Agricultural Research Services, United States Department of Agriculture, Davis, California, United States; ^2^Obesity and Metabolism Research Unit, Western Human Nutrition Research Center, Agricultural Research Services, United States Department of Agriculture, Davis, California, United States; ^3^Department of Nutrition, University of California, Davis, California, United States

**Keywords:** cortisol, fasting, HDL, monocyte, postprandial

## Abstract

Monocytes are innate immune cells that are continuously produced in bone marrow which enter and circulate the vasculature. In response to nutrient scarcity, monocytes migrate back to bone marrow, where, upon refeeding, they are rereleased back into the bloodstream to replenish the circulation. In humans, the variability in monocyte behavior in response to fasting and refeeding has not been characterized. To investigate monocyte dynamics in humans, we measured blood monocyte fluctuations in 354 clinically healthy individuals after a 12-h overnight fast and at 3 and 6 h after consuming a mixed macronutrient challenge meal. Using cluster analysis, we identified three distinct monocyte behaviors. *Group 1* was characterized by relatively low fasting monocyte counts that markedly increased after consuming the test meal. *Group 2* was characterized by relatively high fasting monocyte counts that decreased after meal consumption. *Group 3*, like *Group 1*, was characterized by lower fasting monocyte counts but increased to a lesser extent after consuming the meal. Although monocyte fluctuations observed in *Groups 1* and *3* align with the current paradigm of monocyte dynamics in response to fasting and refeeding, the atypical dynamic observed in *Group 2* does not. Although generally younger in age, *Group 2* subjects had lower whole body carbohydrate oxidation rates, lower HDL-cholesterol levels, delayed postprandial declines in salivary cortisol, and reduced postprandial peripheral microvascular endothelial function. These unique characteristics were not explained by group differences in age, sex, or body mass index (BMI). Taken together, these results highlight distinct patterns of monocyte responsiveness to natural fluctuations in dietary fuel availability.

**NEW & NOTEWORTHY** Our study composed of adult volunteers revealed that monocyte dynamics exhibit a high degree of individual variation in response to fasting and refeeding. Although circulating monocytes in most volunteers behaved in ways that align with previous reports, many exhibited atypical dynamics demonstrated by elevated fasting blood monocyte counts that sharply decreased after meal consumption. This group was also distinguished by lower HDL levels, reduced postprandial endothelial function, and a delayed postprandial decline in salivary cortisol.

## Graphical abstract

**Figure:**
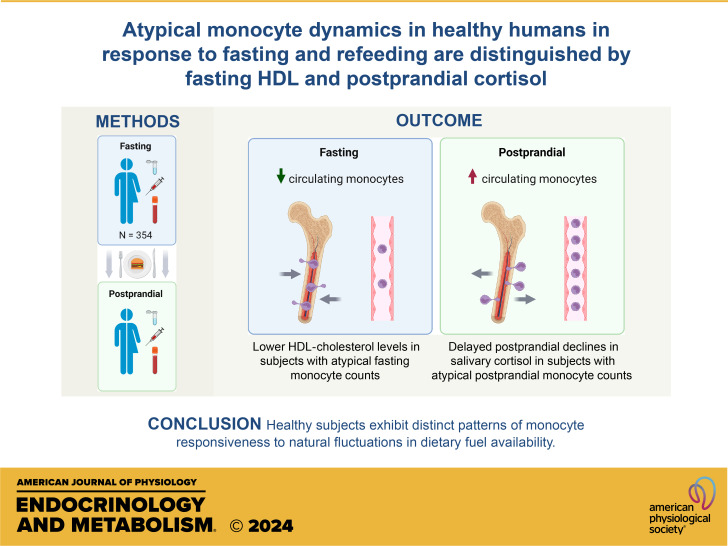

## INTRODUCTION

Monocytes are a versatile and heterogenous population of innate immune cells that play a critical role in the response to infection and tissue injury (1). Originating from myeloid precursor cells in bone marrow, monocytes enter and circulate the vasculature as classical monocytes. Although classical monocytes can extravasate into tissue to become macrophages, some undergo differentiation into non-classical monocytes that maintain vascular integrity by surveilling endothelium and scavenging luminal debris (2). Owing to their relatively short half-lives, massive daily production in bone marrow, and reliance on myelopoiesis for replenishment, monocytes are thought to be an extremely energetically costly cell type (3).

The body’s ability to maintain metabolic homeostasis relies on multiorgan coordinated control of available fuels. Natural fluctuations in dietary fuel availability throughout the day can cause significant oscillations in circulating immune cell populations, particularly monocytes (3–5). In recent years, several studies have investigated how immune cell distributions respond to changes in nutrient availability (3, 4, 6, 7). These reports show that in response to fasting when nutrient availability is low, peripheral monocytes migrate back to bone marrow to conserve energy and extend their lifespan in a protective environment. This “hiding out” strategy may allow the body to conserve resources while prioritizing essential functions. Upon refeeding, monocytes are remobilized back into the bloodstream to replenish the circulation (3). Although mechanisms underlying monocyte dynamics in response to fasting and refeeding are not fully characterized, studies in rodents have shown that the central nervous system (CNS) plays an important role in orchestrating monocyte shifts between circulation and bone marrow via activation of the hypothalamic-pituitary-adrenal axis (HPA) (3). Additional factors shown to influence monocyte dynamics include age (7), high-density lipoprotein cholesterol (HDL-C) (8–10), and sleep patterns (11, 12).

Although monocytes are critical for maintaining health, heightened myelopoiesis and elevated circulating monocyte numbers are associated with the progression of chronic disease including certain types of cancer (13, 14), fibrotic diseases (15), and atherosclerosis and cardiovascular disease (16–18). In subjects without preexisting carotid atherosclerosis, monocyte count was found to be an independent predictor of future plaque formation (19). Although peripheral monocytes are a double-edge sword, identifying factors that affect their dynamics during fasting and refeeding could have important health implications for specific populations. Therefore, to investigate monocyte dynamics in humans, we measured monocyte fluctuations in a cohort of 354 clinically healthy individuals after a 12-h overnight fast and at 3 and 6 h after consuming a mixed macronutrient meal. Although monocyte dynamics in response to fasting and refeeding generally aligned with previous reports, we identified a minor population of study participants who exhibited atypical dynamics prompting us to identify factors that might help explain this atypical pattern of nutrition-related fluctuation in circulating monocytes. As recent studies have attributed changes in monocyte distribution to metabolic fluctuations in response to fasting and refeeding (3, 4), we hypothesized that the atypical dynamics observed in volunteers may be due to factors related to nutrient availability and energy metabolism.

## METHODS

### Study Participants and Dietary Challenge

Study participants were from the United States Department of Agriculture (USDA) Nutritional Phenotyping Study that included healthy men and women, aged 18–66 yr with a normal to obese body mass index (BMI) of 18–44 kg/m^2^ living near Davis, CA, beginning in May 2015 (20). Men and women were recruited to fill nine bins within sex, to balance BMI and age, using three BMI categories (<25, 25–29, and 30–44 kg/m^2^) within each age category (18–33, 34–49, and 50–65 yr). Participants were excluded if they had high blood pressure (systolic blood pressure >140 mmHg or diastolic blood pressure >90 mmHg) when measured on-site or if they had any active chronic disease requiring daily medication, including, but not limited to, diabetes mellitus, cardiovascular disease, cancer, gastrointestinal disorders, kidney disease, liver disease, bleeding disorders, asthma, autoimmune disorders, hypertension, or osteoporosis. Participants were also excluded if they were pregnant or lactating, had recently undergone minor surgery, recently received antibiotic therapy, had been hospitalized in the past 4 wk, or had major surgery in the past 16 wk. The study included two visits to the USDA—Western Human Nutrition Research Center (WHNRC) scheduled within a period of 10–14 days. On *visit 1*, subjects were provided informed consent and screened to ensure volunteers fell within the designed ranges for the study. *Visit 2* entailed consumption of a challenge meal. The night before *visit 2*, subjects were provided a high-carbohydrate meal (17% kcal from fat, 77% kcal from carbohydrate, and 7.5% kcal from protein) that they were instructed to consume before 7:00 PM. Subjects arrived fasted (12 h) the next morning, and fasting blood was collected before ingestion of a high-fat liquid challenge meal (60% kcal from fat, 25% kcal from carbohydrates, and 15% kcal from protein). Additional details of the standardized high carbohydrate dinner and high-fat challenge meal are contained in a separate report (21). Postprandial blood was drawn at 3 and 6 h after consumption of the challenge meal. Ethnicity of the study subjects was self-reported by subjects using a demographic questionnaire and is presented in a separate report (22). The study was registered at clinicaltrials.gov (identifier: NCT02367287) and received ethical approval from the University of California, Davis, Institutional Review Board. All participants provided written informed consent and received monetary compensation for their participation. Data were stored using the Research Electronic Data Capture (REDCap) application hosted by the University of California Davis Health System Clinical and Translational Science Center.

### White Blood Cell and Monocyte Counts

White blood cell and monocyte counts were measured using complete blood count (CBC) with differential. During the 4-yr recruitment period from June 2015 through July 2019, the CBC analyses were performed using whole blood (treated with EDTA as an anticoagulant) in the UC Davis Health, Department of Pathology and Laboratory Medicine Clinical Laboratory using a Beckman Coulter LH750/780 (before October 2016) or a Beckman Coulter DxH800 automated hematology analyzer, with the exception that 12 samples early in the study (before August 14, 2015) were analyzed on an Abbott Cell-Dyn 322 analyzer at the WHNRC.

### Monocyte Subset Analysis by Flow Cytometry

Monocyte subsets at fasting and postprandial time points were analyzed using 100 µL of whole blood collected into EDTA-treated tubes mixed with pretitrated volumes of the following antibodies in BD Brilliant stain buffer (Cat. No. 563794; BD Biosciences): CD45-BV786 (Cat. No. 563716; BD Biosciences), CD91-PE (Cat. No. 550497; BD Biosciences), CD14-BUV395 (Cat. No. 563561; BD Biosciences), and CD16-BV421 (Cat. No. 562878; BD Biosciences). Following a 20-min incubation at room temperature, 1× BD FACS Lysing Solution (Cat. No. 349202; BD Biosciences) was added to the whole blood/antibody mixture and incubated at room temperature for an additional 10 min. Cells were washed twice with cold wash/stain buffer [containing 0.1% BSA (wt/vol), 0.05% NaN3 (wt/vol) in PBS] then analyzed using an LSR Fortessa flow cytometer (BD Biosciences) configured with blue (488 nm), red (640 nm), violet (405 nm), and UV lasers (355 nm). Data were collected using FACSDiva and analyzed using FlowJo v.10.6.1 software (BD Biosciences). The gating strategy for monocyte subset analysis is contained in a separate report (7).

### Clinical Parameters

Fasting and postprandial blood was collected, and serum or plasma was obtained by centrifugation at 1300 *g* at 4°C for 10 min. Lipid-related markers, including triglycerides, total cholesterol, HDL-cholesterol (HDL-C), and LDL-cholesterol (LDL-C), were measured using a Cobas Integra 400/800 kit (Roche, Indianapolis, IN), a Cobas CHOL2 kit (Roche), a Cobas HDL-C plus 3rd generation kit (Roche), and a Cobas LDLC3 kit (Roche), respectively. All assays were completed on an auto-analyzer, Cobas Integra 400+ instrument (Roche). Plasma glucose concentrations were measured using Glucose HK Gen.3 kits (Roche) conducted on the Cobas Integra 400+ instrument (Roche). Serum insulin levels were determined by Elecsys Insulin kits running on a Cobas e411 analyzer (Roche). Blood pressure was measured using a standard blood pressure cuff placed on one arm.

### Salivary Cortisol

Relative concentrations of circulating free cortisol are reliably estimated by salivary cortisol concentrations (23, 24). We examined salivary cortisol concentrations at fasting and 30, 60, and 90 min after consumption of the test meal. Salimetrics oral swabs were used to collect Saliva samples. Research volunteers placed a swab in their mouth for 1–2 min and then deposited the swab into sample tubes. Swab samples were immediately centrifuged to collect and aliquot saliva. Aliquots were stored at −80°C until being assayed for cortisol. Salivary cortisol concentrations were determined using an ELISA (expanded-range high-sensitivity salivary cortisol kit; Salimetrics). Cortisol concentrations ranging from 0.193 to 82.77 nmol/L (0.007–3.0 μg/dL) can be detected by this assay, which has a typical intraassay coefficient of variation (CV) of 3.5% and interassay CV of 5.1%.

### Resting Metabolic Rate and Substrate Utilization

Metabolic rate (energy expenditure) and substrate oxidation were estimated from indirect calorimetry. A ParvoMedics (Sandy, UT) TrueOne 2400 metabolic cart was used to collect respiratory gases. Before each test, the metabolic cart was calibrated as described in the study of Richardson et al. (25). The metabolic cart procedure was conducted in a dark, quiet room with study volunteers lying down throughout the procedure. After a 10-min resting period at each timepoint, respiratory gases were collected for 20 min before (fasting) the test meal and 0.5, 3, and 6 h after consuming the test meal. To allow the participant to acclimate to the protocol, data from the first 5 min were excluded from the analysis (25). The Weir equation (kcal/min = (3.94 × Vo_2_) + (1.11 × Vco_2_)) (26) was used to estimate resting metabolic rate (kcal/min). The nitrogen correction was ignored since nitrogen excretion is minimal in such a short period of time (25). Total carbohydrate and fat oxidation were calculated using the following equations assuming protein oxidation was close to zero for this brief time period (27): carbohydrate oxidation (g/min) = (4.55 × Vco_2_ L/min) − (3.21 × Vo_2_ L/min) and fat oxidation (g/min) = (1.67 × Vo_2_ L/min) − (1.67 × Vco_2_ L/min).

### Plasma Immune Markers Measured by Multiplexed MSD Assay

The concentrations of plasma proteins (µg/L) were measured in plasma using MSD assay kits and the MSD sector imager 2400 (MESO Scale Discovery). EDTA plasma was used for proteins (CRP using the Vplex Vascular Injury Panel 1 with samples diluted 1:1,000; CCL2 using the Vplex Custom Human Biomarker Chemokine Panel 1 with samples diluted 1:4; TNF-α, IL-1β, and IL-6 using the Vplex Custom Human Biomarker Proinflammatory Panel 1 with samples diluted 1:2). Three levels of lyophilized controls were used on each plate to assess plate-to-plate variation. Mean concentrations (µg/L) of duplicate wells were used for analysis.

### Vascular Function

Peripheral vascular function was estimated using an Itamar Medical (Ltd., Caesarea, Israel) EndoPat system, as reported (28). Both endothelial function [reactive hyperemia index (RHI)] and arterial stiffness [augmentation index (AI)] were used to indicate vascular function. Reactive hyperemia is an increase in blood flow following transient arterial occlusion and is dependent on the ability of blood vessels to dilate properly; a higher RHI indicates better endothelial function. A lower AI indicates less arterial stiffness or more arterial flexibility.

### Statistical Analysis

**Figure 1.**
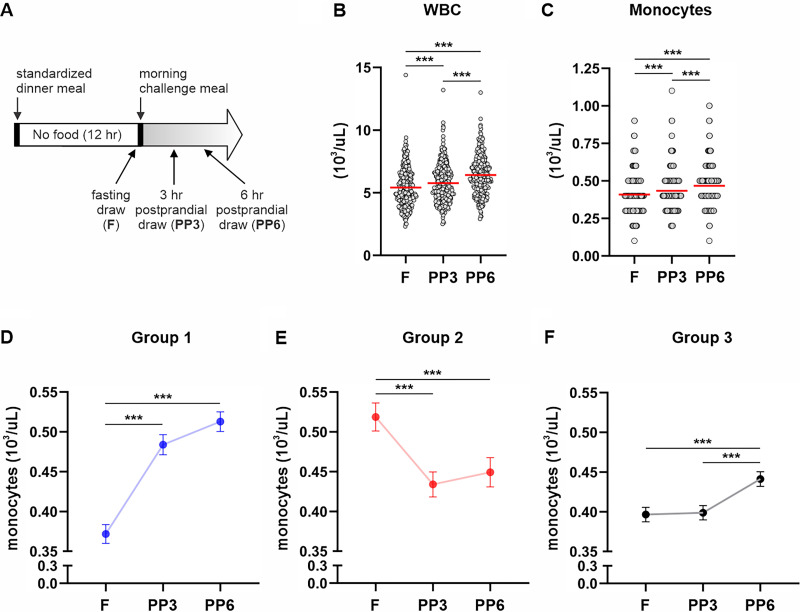
Monocyte dynamics during fasting and refeeding. *A*: schematic of the study design. Absolute circulating WBC (*B*) and monocyte (*C*) counts after 12-h overnight fast and at 3 and 6 h after consuming a mixed macronutrient meal. Monocyte dynamics during fasting and refeeding for subjects in *Group 1* (*D*), *Group 2* (*E*), and *Group 3* (*F*). Mean and individual data points are shown in *B* and *C*, whereas means ± SE are presented in *D*–*F*. Repeated-measures analysis was used to identify a significant effect of time in *B*–*F* (Time, *P* < 0.0001) that was followed up with individual *t* tests to determine differences between time points; ***P < 0.001. F, fasting; PP, postprandial; PP3, 3-h postprandial; PP6, 6-h postprandial; WBC, white blood cell.

Statistical analyses were performed using SAS for Windows, release 9.4 (Cary, NC) and GraphPad Prism 10, v.10.2.1. Cluster analysis (Proc Fastclus) was conducted to determine unique clusters (groups) of monocyte concentration patterns in study participants (*n* = 354). The 3- and 6-h changes in postprandial monocyte concentration variables were standardized using the Proc Stdize procedure before being subjected to the cluster analysis. From this analysis, three unique clusters were identified, as shown in Fig. 1. One cluster included a minor population of study participants that exhibited an atypical fasting and postprandial response pattern. Therefore, to identify factors that might help explain this atypical pattern of nutrition-related fluctuation in circulating monocytes, we separately compared this cluster with each of the other two clusters. A mixed-model procedure (Proc Mixed) adjusted for repeated measures was used to test for differences between monocyte clusters in fasting and postprandial salivary cortisol concentrations and energy metabolism [metabolic rate, carbohydrate and fat oxidation rates, circulating concentrations of glucose, free fatty acids (NEFA), triglycerides, total cholesterol, HDL-cholesterol, and LDL-cholesterol]. This analysis was conducted to test for the main effects of cluster and time, and for a cluster-by-time interaction. A statistically significant monocyte cluster-by-time interaction was followed up with *t*-tests at each timepoint to determine the nature of the interaction. With a nonsignificant interaction effect, a main effect of cluster indicated time-independent differences between clusters. General linear models (Proc GLM) were also used to test for cluster differences in fasting variables (Table 1) and vascular function (RHI and AI). Sex, age category, and BMI category were included as independent variables in all statistical models. Because metabolic rate influences substrate oxidation, models examining carbohydrate and fat oxidation also included metabolic rate as an independent variable. Sample sizes for analysis on dependent variables ranged from *n* = 354 to *n* = 327. For all statistical analyses a *P* value of ≤0.05 was considered statistically significant. Final assembly and preparation of all figures were done using CorelDRAW Essentials 2021 (Corel Corporation, Ottawa, Canada).

**Table 1.** Fasting characteristics of Groups 2 and 1 and Groups 2 and 3

	*Group 2*	*Group 1*	*P* Value	*Group 3*	*P* Value
*n*	59	123		172	
Male, %	50.9	40.2	ns	52.9	ns
Age, yr	34.0 (19–63)	40.8 (18–65)	0.0013	41.9 (19–66)	0.0002
BMI, kg/m^2^	27.65 (17.92–42.87)	27.59 (18.04–43.31)	ns	26.76 (18.40–41.74)	ns
Energy intake, kcal/day	2,372 (958.0–6,115)	2,018 (750.6–5,266)	0.0124	2,085 (704.7–4,807)	0.0307
WBC, 10^3^/µL	6.35 (3.4–14.4)	5.36 (2.5–9.4)	<0.0001	5.13 (2.3–8.8)	<0.0001
Monocytes, 10^3^/µL	0.52 (0.3–0.9)	0.37 (0.1–0.8)	<0.0001	0.40 (0.2–0.9)	<0.0001
TGs, mg/dL	102.3 (34–405)	98.8 (37–421)	ns	93.1 (30–219)	ns
NEFAs, mEq/L	0.34 (0.06–0.78)	0.32 (0.07–0.78)	ns	0.31 (0.07–0.69)	ns
Cholesterol, mg/dL	172.5 (111.8–263.1)	179.1 (103.3–261.6)	ns	172.0 (88.5–316.8)	ns
HDL-C, mg/dL	50.0 (30.8–80.5)	57.1 (23.8–109.4)	0.0051	55.8 (27.5–116.0)	0.0155
LDL-C, mg/dL	110.9 (60.7–187.5)	112.2 (52.5–200.8)	ns	107.5 (38.5–226.0)	ns
Insulin, pmol/L	8.88 (1.73–28.61)	10.04 (2.43–40.92)	ns	7.85 (1.38–28.98)	ns
Glucose, mg/dL	92.87 (62.4–111.6)	94.42 (77.4–132.5)	ns	94.36 (69.4–139.5)	ns
IL-6, pg/mL	0.78 (0.19–2.52)	0.75 (0.03–5.15)	ns	0.66 (0.12–2.39)	ns
TNF-α, pg/mL	2.20 (1.04–5.55)	2.13 (0.75–5.11)	ns	2.17 (0.35–7.90)	ns
IL-1β, pg/mL	0.067 (0.000–0.231)	0.079 (0.010–0.262)	ns	0.073 (0.004–0.297)	ns
CRP, ng/mL	4,076 (169–32,686)	4,315 (62–75,352)	ns	3,045 (50–38,059)	ns
CCL2, pg/mL	97.6 (44.6–230.0)	95.1 (39.1–269.6)	ns	101.7 (44.4–333.7)	ns
Sal. cortisol	8.81 (2.43–23.68)	7.93 (2.35–27.05)	ns	8.22 (0.11–22.99)	ns

Values are means and range (in parentheses) for each quantitative parameter. Outcomes shown in this table are not statistically adjusted for age, sex, or BMI. BMI, body mass index; CCL2, C-C motif chemokine ligand 2; CRP, C-reactive protein; HDL-C, high-density lipoprotein cholesterol; LDL-C, low-density lipoprotein cholesterol; NEFAs, nonesterified fatty acids; Sal. cortisol, salivary cortisol; TGs, triglycerides; WBC, white blood cell.

## RESULTS

We investigated monocyte dynamics in a cohort of 354 clinically healthy individuals in response to a 12-h overnight fast and at 3 and 6 h after consuming a mixed macronutrient meal. A schematic of the study design is presented in Fig. 1*A*. In agreement with previous reports (3, 5, 29, 30) circulating WBC and monocyte counts at fasting significantly increased in response to refeeding (Fig. 1, *B* and *C*). To assess individual variability of monocyte behaviors, we used cluster analysis to group subjects based on blood monocyte counts at fasting and after refeeding. Cluster analysis identified three groups. Relative to fasting counts, monocytes in *Group 1* increased at 3 h after consuming a mixed macronutrient meal and remained elevated at 6 h (Fig. 1*D*). In *Group 2*, blood monocyte counts sharply decreased at 3 h after meal consumption and changed little at 6 h (Fig. 1*E*). In *Group 3*, peripheral monocyte counts did not differ between fasting and the 3-h postprandial time point but moderately increased at 6 h (Fig. 1*F*). Taken together, monocyte dynamics in *Groups 1* and *3* appeared to behave in ways that align with previous reports (3, 5, 29) that can be generally characterized by moderate to strong postprandial increases. Alternatively, we identified an atypical dynamic in *Group 2* that comprised 17% of the study cohort and that was characterized by a strong postprandial decline in monocyte counts.

**Figure 2.**
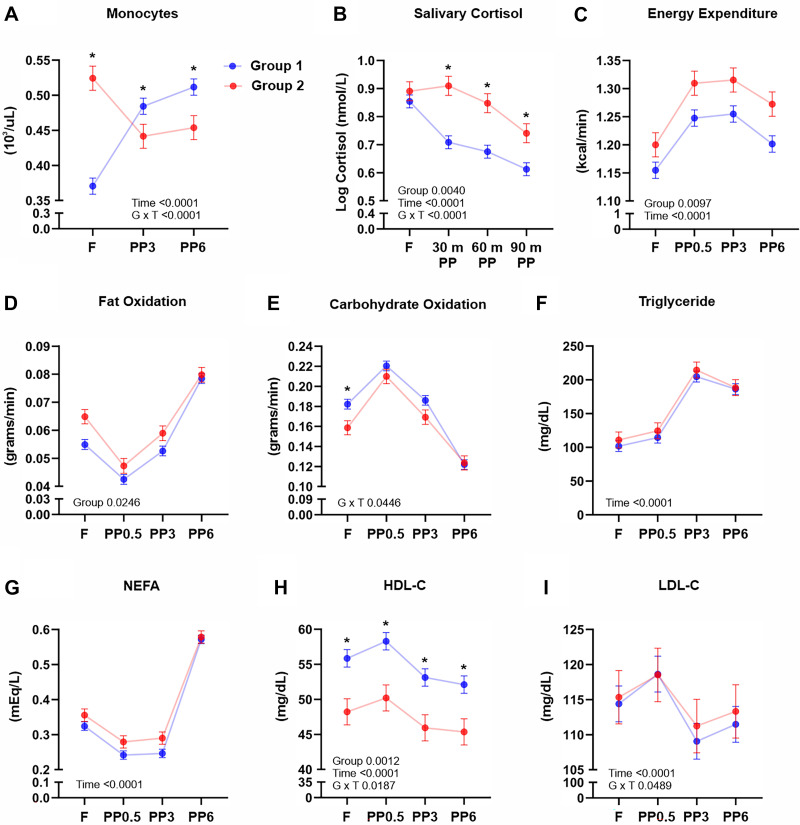
Comparisons between *Group 1* and *Group 2* during fasting and refeeding. Comparison of circulating monocyte counts (*A*), salivary cortisol (*B*), energy expenditure (*C*), fat oxidation (*D*), carbohydrate oxidation (*E*), triglyceride levels (*F*), nonesterified fatty acids (*G*), HDL-C (*H*), and LDL-C (*I*) between *Group 1* and *Group 2* subjects during fasting and refeeding. Values depicted are means ± SE adjusted for sex, age, and BMI. *Significant differences between groups at time point as determined by *t* test only when a significant group-by-time interaction was observed; *P < 0.05. BMI, body mass index; F, fasting; PP, postprandial; PP0.5, 0.5-h postprandial; PP3, 3-h postprandial; PP6, 6-h postprandial.

As recent studies have attributed changes in monocyte distribution to metabolic fluctuations in response to fasting and refeeding (3, 4), we hypothesized that the atypical dynamic observed in *Group 2* subjects may be due to factors related to nutrient availability and energy metabolism. We applied a mixed model adjusted for repeated measurements to test for a main effect of group, main effect of time, and a group-by-time interaction. A main effect of group indicates an overall average and time-independent difference between groups. A main effect of time suggests overall group-independent differences across time. A group-by-time interaction supports group differences that vary with time or time differences that depend on group. Consistent with our assessment in Fig. 1, repeated-measures analyses of monocyte counts between *Group 2* and the more typical *Group 1* indicated a significant group-by-time interaction (*P* = 0.0001). As indicated in Fig. 2*A*, fasting monocytes were higher in *Group 2* and then sharply declined 3 h postprandially. In comparison, circulating monocyte concentrations in *Group 1* increased 3 and 6 h above fasting levels and after the test meal was consumed. As shown in Fig. 2*B* and supported by a significant group-by-time interaction (*P* = 0.0001), *Groups 1* and *2* also differed in postprandial, but not fasting, salivary cortisol concentrations. We found an overall main effect of group, but not group-by-time interaction, for resting energy expenditure (group, *P* = 0.0097) and fat oxidation (group, *P* = 0.0246), suggesting that, on average and independent of time, these metabolic variables were generally higher in *Group 2* compared with *Group 1* (Fig. 2, *C* and *D*). Carbohydrate oxidation was significantly lower in *Group 2*, but only at the fasting time point, which is supported by a group-by-time interaction (*P* = 0.0446, Fig. 2*E*). Although the test meal induced significant (Time, *P* = 0.0001) shifts in plasma concentrations of nonesterified fatty acids (NEFA), this metabolite did not differ between *Groups 1* and *2* (Fig. 2*F*). Compared with *Group 1*, *Group 2* had higher circulating LDL-cholesterol and lower HDL-cholesterol concentrations, but a significant (*P* < 0.05) group-by-time interaction suggests a time-dependent nature of these differences (Fig. 2, *G* and *H*). Circulating concentrations in insulin, glucose, triglycerides, and total cholesterol did not differ between *Groups 1* and *2* (*P* > 0.05). Table 1 displays results from additional analyses of fasting variables between *Groups 1* and *2*. These results are supportive of the above presented results derived from the repeated-measures analyses, but also showed age (*P* = 0.0013), habitual energy intake (*P* = 0.0124), and fasting white blood cell counts (*P* < 0.0001) to differ between *Groups 1* and *2*.

**Figure 3.**
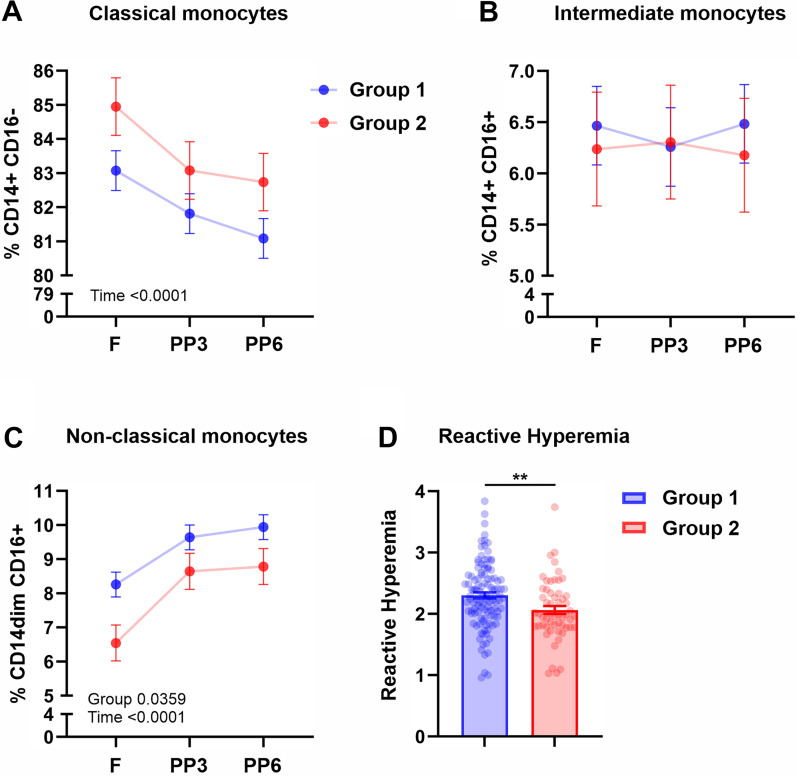
Monocyte subsets and reactive hyperemia in *Group 1* and *Group 2*. Frequencies of classical (*A*), intermediate (*B*), and non-classical (*C*) monocytes after 12-h overnight fast and at 3 and 6 h after consuming a mixed macronutrient meal. *D*: postprandial reactive hyperemia peripheral arterial tonometry index. Values depicted are means ± SE adjusted for sex, age, and BMI; **P < 0.01. BMI, body mass index; F, fasting; PP, postprandial; PP3, 3-h postprandial; PP6, 6-h postprandial.

Next we examined frequencies of classical, intermediate, and non-classical monocyte subsets after a 12-h overnight fast and at 3 and 6 h after consuming the mixed macronutrient test meal. Repeated-measures analyses revealed that both *Groups 1* and *2* had postprandial decreases in classical monocytes (*P*_time_ = 0.0001, Fig. 3*A*), but the frequency of this monocyte subset did not differ between these groups at any timepoint (*P* > 0.05). No effects were found for intermediate monocytes (Fig. 3*B*), but a main effect of group (*P* = 0.0359) and time (*P* = 0.0001) was observed for non-classical monocytes, suggesting that, although non-classical monocytes showed postprandial increases in both *Groups 1* and *2*, *Group 1* systematically had a higher frequency of non-classical monocytes across all timepoints (Fig. 3*C*). Considering that non-classical monocytes are important for surveilling endothelium and maintaining vascular integrity that is known to be impaired following consumption of a single high-fat meal (31–33), we measured peripheral microvascular endothelial function by reactive hyperemia. Compared with *Group 1*, *Group 2* had a lower reactive hyperemia index (*P* = 0.0037, Fig. 3*D*) although the augmentation index, which is an indicator of arterial stiffness (34), did not differ between these groups (*P* = 0.097).

**Figure 4.**
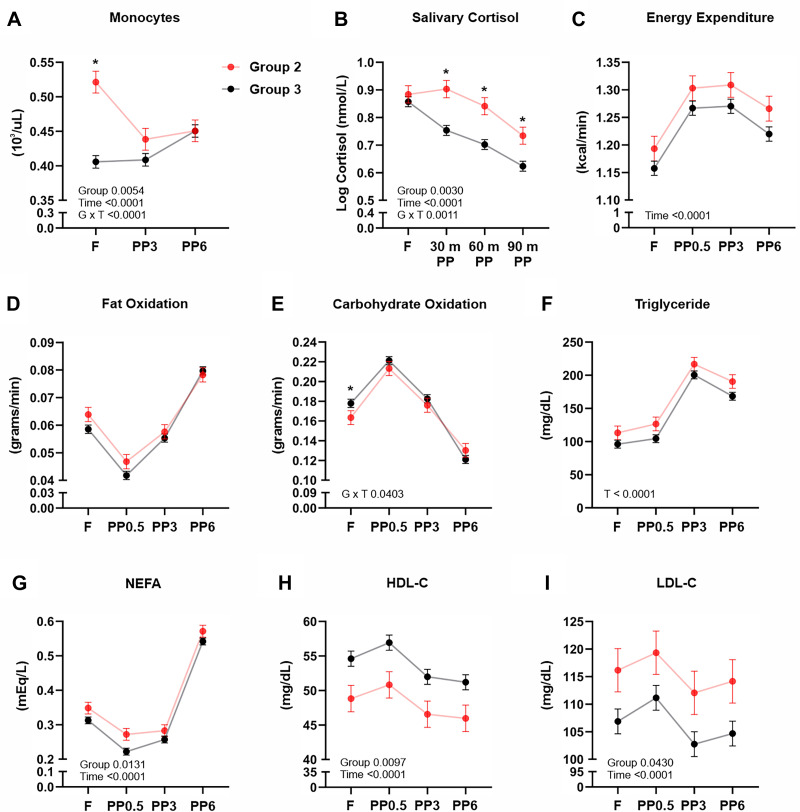
Comparisons between *Group 2* and *Group 3* during fasting and refeeding. Comparison of circulating monocyte counts (*A*), salivary cortisol (*B*), energy expenditure (*C*), fat oxidation (*D*), carbohydrate oxidation (*E*), triglyceride levels (*F*), nonesterified fatty acids (*G*), HDL-C (*H*), and LDL-C (*I*) between *Group 2* and *Group 3* subjects during fasting and refeeding. Values depicted are means ± SE adjusted for sex, age, and BMI. *Significant differences between groups at time point as determined by *t* test only when a significant group-by-time interaction was observed; *P < 0.05. BMI, body mass index; F, fasting; PP, postprandial; PP0.5, 0.5-h postprandial; PP3, 3-h postprandial; PP6, 6-h postprandial.

Repeated-measures analyses of monocyte counts between *Group 2* and *Group 3*, which was also characterized by a more typical, yet moderate, postprandial increase in monocyte counts, showed a significant group-by-time interaction (*P* = 0.0001). As indicated in Fig. 4*A*, fasting monocytes were lower in *Group 3*, with no increase at 3 h, but moderate increase at 6 h postprandially. Monocyte counts only differed between *Groups 2* and 3 at the fasting timepoint. As shown in Fig. 4*B* and supported by a significant group-by-time interaction (*P* = 0.0001), *Groups 2* and *3* also differed in postprandial, but not fasting, salivary cortisol concentrations. We found an overall main effect of time, but not group, for resting energy expenditure (Time, *P* = 0.0001, Fig. 4*C*). Carbohydrate oxidation was significantly lower in *Group 2*, but only at the fasting time point, which is supported by a group-by-time interaction (*P* = 0.0403, Fig. 4*E*). As shown in Fig. 4*F*, the test meal induced significant (Time, *P* = 0.0001) shifts in plasma concentrations of NEFAs in both groups, but NEFA was generally higher in *Group 2* (Group, *P* = 0.0131). Compared with *Group 3* and as suggested by the main effect of group, *Group 2* had time-independent lower HDL-cholesterol concentrations (Group, *P* = 0.0097) and higher circulating LDL-cholesterol concentrations (*P* = 0.0430). As suggested by a time effect (*P* = 0.0001), both groups showed postprandial increases at 0.5 h and then subsequent decreases at 3 h in HDL- and LDL-cholesterol (Fig. 4, *G* and *H*). Circulating concentrations in insulin, glucose, triglycerides, and total cholesterol did not differ between *Groups 2* and *3* (*P* > 0.05). Table 1 displays results from additional analyses of fasting variables between *Groups 2* and *3*. These results are supportive of the above presented results derived from the repeated-measures analyses, but also showed age (*P* = 0.0002), habitual energy intake (*P* = 0.0307), and fasting white blood cell counts (*P* < 0.0001) to differ between *Groups 2* and *3*.

**Figure 5.**
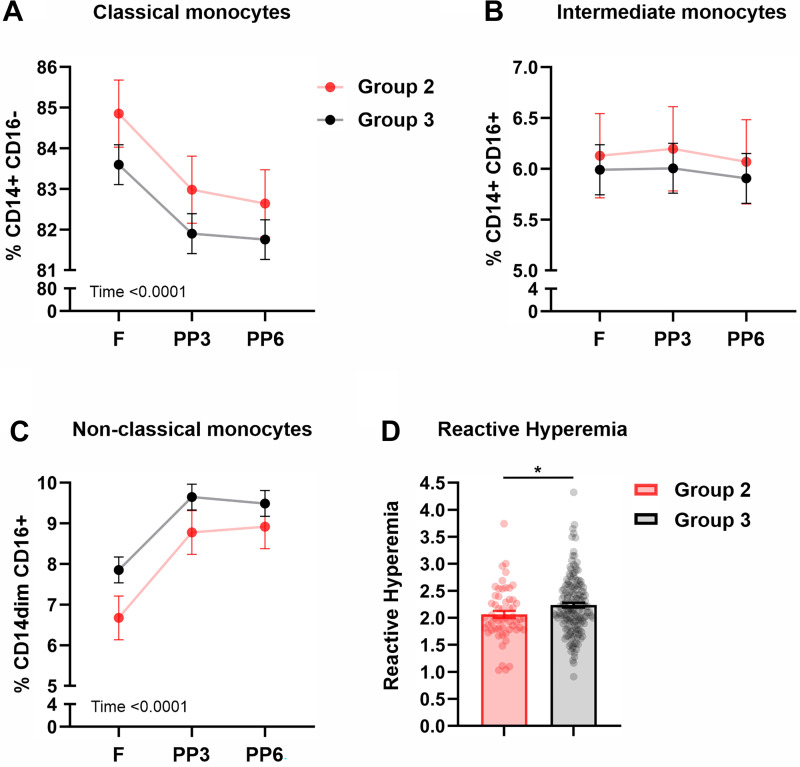
Monocyte subsets and reactive hyperemia in *Group 2* and *Group 3*. Frequencies of classical (*A*), intermediate (*B*), and non-classical (*C*) monocytes after 12-h overnight fast and at 3 and 6 h after consuming a mixed macronutrient meal. *D*: postprandial reactive hyperemia peripheral arterial tonometry index. Values depicted are means ± SE adjusted for sex, age, and BMI; *P < 0.05. BMI, body mass index; F, fasting; PP, postprandial; PP3, 3-h postprandial; PP6, 6-h postprandial.

Regarding circulating monocyte subsets, repeated-measures analyses revealed that both *Groups 2* and *3* had postprandial decreases in classical monocytes (*P*_time_ = 0.0001, Fig. 5*A*), whereas non-classical monocytes increased postprandially in both groups (*P*_time_ = 0.0001, Fig. 5*C*). The frequency of the intermediate monocytes did not change with time (*P* > 0.05) and did not differ between *Groups 2* and *3* (*P* > 0.05). Compared with *Group 3*, *Group 2* had a lower reactive hyperemia index (*P* = 0.0324, Fig. 3*D*), but the augmentation index did not differ between these groups (*P* = 0.8446).

## DISCUSSION

We investigated monocyte dynamics in response to fasting and refeeding in healthy adults recruited to a cross-sectional Nutritional Phenotyping Study. Using cluster analysis, we identified three distinct monocyte behaviors in response to fasting and refeeding. Dynamics in cluster *Groups 1* and *3*, which collectively comprised 83% of study subjects, behaved in ways that align with previous reports that showed relatively low fasting monocyte counts followed by increased counts in the postprandial state (4, 5, 29). In contrast, *Group 2* exhibited an atypical pattern that resembled the irregular monocyte fluctuations previously reported in healthy individuals (4). Taken together, these results in clinically healthy subjects highlight distinct patterns of monocyte responsiveness to natural fluctuations in dietary fuel availability.

In addition to exhibiting elevated monocyte counts at fasting, *Group 2* subjects were generally younger in age, had higher habitual energy intakes, exhibited lower carbohydrate oxidation rates, and had lower HDL levels compared with subjects in *Groups 1* and *3*. Although HDL is well recognized for its role in reverse cholesterol transport to the liver, it also regulates circulating monocyte counts through controlling myelopoiesis in bone marrow (8, 10). In a steady state, monocytes and other immune cells are continuously produced from precursor cells in bone marrow that are ultimately derived from a small pool of self-renewing pluripotent hematopoietic stem cells (HSCs) (35). In response to various stimuli, HSCs undergo lineage commitment steps while proliferating and differentiating. Recent studies found that elevated plasma and intracellular cholesterol enhance HSC expansion and myeloid skewing, whereas HDL, through promoting cholesterol efflux from HSCs, suppresses proliferation and monocytic skewing (8, 14, 36). This effect of HDL aligns with our findings of lower fasting blood monocytes in participants with higher circulating HDL concentrations. In addition to undergoing developmental regulation in bone marrow, circulating immune cell behavior can be influenced by fluctuating endocrine cues and metabolic signals during transitions between feeding and fasting, and vice versa (3, 4, 6). In response to fasting, peripheral monocytes undergo reverse mobilization, migrating from blood and peripheral tissues, including lung, liver, and spleen, back to bone marrow (3, 4). Although mechanisms and implications are only partly understood, studies suggest monocytes use the bone marrow as a haven to tide off periods of metabolic adversity (4, 6, 37). Although it is currently unclear why bone marrow functions as a hibernating tissue of choice for monocytes, it has been suggested that it may provide a more energy-rich tissue microenvironment than cells would otherwise encounter in circulation during the fasted state (38). However, a recent report showed that monocytes isolated from bone marrow of fasting mice exhibit quiescent metabolic phenotypes as demonstrated by reduced basal and maximal oxygen consumption and reduced basal extracellular acidification compared with bone marrow monocytes isolated from fed mice (4). Therefore, we propose an alternative hypothesis for reverse mobilization of monocytes in response to reduced nutrient availability. Owing to their high energetic demands and immense numbers in circulation, mobilizing peripheral monocytes back to bone marrow during periods of nutrient scarcity may ensure that reduced levels of circulating glucose remain available for utilization by tissues with an obligatory requirement for glucose.

Although monocyte fluctuations observed in most study volunteers align well with the current paradigm of monocyte dynamics in response to fasting and refeeding, we cannot explain the atypical dynamic observed in *Group 2*. Lower whole body carbohydrate oxidation rates at fasting demonstrated by *Group 2* subjects may suggest reduced reliance on this fuel type (e.g., less utilization of glycogen reserves) to maintain glucose availability. With a net balance of higher whole body fat-to-carbohydrate oxidation at fasting, the body’s need to mobilize energy-consuming monocytes in circulation back to bone marrow to spare glucose would be minimal. Furthermore, although circulating monocyte counts are generally lowest during early morning hours (39–41), a single night of sustained wakefulness in healthy subjects was shown to increase morning monocyte counts (39) and alter subset frequencies (40) compared with subjects who underwent a regular night of nocturnal sleep. Although we cannot overlook the contribution of sleep patterns on fasting monocyte counts in our study cohort, we think its impact on elevated fasting monocyte counts observed in *Group 2* subjects is minimal based on the following. First, *Group 2* subjects did not exhibit increased frequencies of non-classical monocytes that were reported in subjects following a night of sustained wakefulness (40). Second, levels of proinflammatory cytokines IL-1β and TNF-ɑ were not elevated in *Group 2* subjects, as were reported in volunteers following a night of sustained wakefulness (39).

In response to refeeding, monocytes that undergo reverse mobilization and migrate back to bone marrow during fasting are remobilized into the bloodstream to replenish the circulation (3). Although neuroendocrine systemic circuits are pivotal in orchestrating immune cell shifts between circulation and bone marrow (3, 42), Janssen et al. (3) recently showed that increases in HPA activity and production of corticosterone (primary glucocorticoid in rodents) facilitate immune cell behavior in response to changes in nutritional state. Cortisol, which is the primary glucocorticoid in humans, is recognized as a critical effector of the physiological adaptation to stress and serves a vital function in regulating carbohydrate, fat, and protein metabolism in both the fed and fasted state (43). In humans, circulating cortisol exhibits a diurnal pattern. It is typically characterized by high concentrations upon waking, followed by a rise that peaks 30 min after awakening and subsequent decline over the day with lowest levels approximately 3–5 h after onset of sleep (43, 44). Later in the sleep cycle when insulin is low, cortisol begins to rise again and promotes gluconeogenesis, lipolysis, and a shift towards lipid utilization in order to meet energy demands and ensure glucose availability (43, 45). These elevations in cortisol are also believed to promote arousal and facilitate a drive to eat (46). After a brief increase upon waking, there is a circadian-driven decline in cortisol levels throughout the day, but consuming breakfast or the first meal of the day augments this decrease in cortisol (47–50). If breakfast or the first meal of the day is not eaten after an overnight fast, the reduction in circulating cortisol concentrations (or corticosterone in rodents) is diminished, which helps sustain energy metabolism and spare glucose utilization during the prolonged fast (47–50). In our study, although salivary cortisol concentrations at fasting were similar between all groups, postprandial levels followed remarkedly different trajectories. In *Groups 1* and *3*, cortisol levels dropped sharply within 30 min and continued to drop at 60 and 90 min after meal consumption. In contrast, cortisol levels in *Group 2* were not reduced at 30 min after meal consumption. And although levels eventually declined, postprandial cortisol levels remained higher at 60- and 90-min timepoints compared with *Groups 1* and *3*. Studies in rodents suggest that increases in glucocorticoids promote a shift of monocytes from circulation to bone marrow through directly binding monocytic glucocorticoid receptor NR3C1 that in turn upregulates expression of chemokine receptor CXCR4 to facilitate migration (3). Consistent with that report, our findings in humans also suggest that higher postprandial cortisol may have contributed to the sharp decrease in postprandial monocytes observed in *Group 2* subjects.

Monocytes, particularly of the non-classical subset, are an important cell type for surveilling endothelium and maintaining vascular integrity whose impairment following consumption of a single high-fat meal is well documented (31–33). The increased frequencies of non-classical monocytes observed in all groups following consumption of the meal may suggest a need for additional vascular surveillance, including patrolling of the endothelium, removing cellular debris, and resolving local inflammation (2). Although proportions of non-classical monocytes increased in all groups, postprandial peripheral microvascular endothelial function, as assessed by reactive hyperemia, was lower in *Group 2* subjects compared with *Groups 1* and *3*. Although all adult volunteers were clinically healthy, lower values of reactive hyperemia are associated with microvascular endothelial dysfunction, which is an early indicator of atherosclerosis (51). Even modest elevations in daily cortisol concentrations can influence cognitive, cardiovascular, metabolic, and immune functions (43–45, 47). Therefore, higher postprandial cortisol concentrations in monocyte *Group 2* may partly explain lower endothelial function in that group. Glucocorticoids can act via glucocorticoid or mineralocorticoid receptors in the vasculature to facilitate vasoconstriction (52, 53). A higher cortisol response to ACTH administration was inversely associated with reactive hyperemia (54), and other studies demonstrated lower vascular endothelial function in patients with Cushing’s syndrome, and impaired microvascular function and lower reactive hyperemia as a consequence of glucocorticoid treatment (55–57).

We acknowledge the limitations associated with this cross-sectional study. The cross-sectional nature of this data analysis is a potential limitation because we cannot assert causality or mechanisms of action. Although we identified physiological and metabolic variables that may, in part, explain human variance in fasting and postprandial fluctuations of circulating monocytes, future experiments should be conducted to help explain mechanisms in humans that mediate nutritional regulation of monocyte behavior. Clinical trials that include dietary manipulations and that monitor monocyte behavior, metabolism, and physiology during the sleep period may help improve our mechanistic understanding of and factors that explain interindividual differences in monocyte responses to fasting and feeding. Although we did not detect changes in frequencies of monocyte subsets between groups using a conventional CD14- and CD16-based flow cytometry panel, newer technologies, including high-parameter cytometry or scRNA-Seq, may be useful for examining frequencies of recently identified monocyte subpopulations such as Slan+CXCR6+ non-classical monocytes that contribute to maintenance of vascular homeostasis (58). Our findings, which support phenotypic differences in nutritional regulation of monocyte dynamics in humans, do warrant further investigation of whether and to what degree cortisol, HDL-cholesterol, and possibly energy homeostasis explain variability in nutritional-related monocyte dynamics.

In summary, our study composed of healthy adult volunteers revealed that monocyte dynamics exhibit a high degree of individual variation in response to fasting and refeeding. Although circulating monocyte counts in most cohort volunteers behaved in ways that align with previous reports, 17% of subjects exhibited atypical dynamics demonstrated by relatively high fasting blood monocyte counts that sharply decreased after meal consumption. This group was also distinguished by lower carbohydrate oxidation rates, higher habitual energy intakes, lower HDL levels, reduced postprandial endothelial function, and a delayed postprandial decline in salivary cortisol. Follow-up studies are clearly needed to determine specific mechanisms differentiating these groups and whether the *Group 2* phenotype displayed in 17% of study volunteers is associated with increased risk for the development of chronic disease.

## ETHICAL APPROVALS

The study was registered on ClinicalTrials.gov (ID: NCT02367287) and received ethical approval from the University of California Davis Institutional Review Board. This study was carried out at the US Department of Agriculture/Agricultural Research Services/Western Human Nutrition Research Center at Davis, CA. Generally, healthy people living near Davis, CA, were invited to participate in this cross-sectional study. Details of the study were explained to and discussed with participants, and those who agreed to the terms of the study provided written informed consent during the first study visit.

## DATA AVAILABILITY

Requests for data from the US Department of Agriculture/Agricultural Research Services (USDA/ARS), Western Human Nutrition Research Center (WHNRC) Nutritional Phenotyping Study used in this analysis should be made via an email to the senior WHNRC author on this publication. Requests will be reviewed quarterly by a committee consisting of the study investigators.

## GRANTS

This work was supported by US Department of Agriculture/Agricultural Research Services (USDA/ARS)/Western Human Nutrition Research Center Project fund 2032–51530-026-00 D.

## DISCLOSURES

No conflicts of interest, financial or otherwise, are declared by the authors.

## AUTHOR CONTRIBUTIONS

R.G.S., C.B.S., and K.D.L. conceived and designed research; R.G.S., C.B.S., and K.D.L. performed experiments; R.G.S. and K.D.L. analyzed data; R.G.S. and K.D.L. interpreted results of experiments; R.G.S. and K.D.L. prepared figures; R.G.S. and K.D.L. drafted manuscript; R.G.S., C.B.S., and K.D.L. edited and revised manuscript; R.G.S., C.B.S., and K.D.L. approved final version of manuscript.
